# Quantification of hemi-hepatic ischemia using real-time multispectral oxygenation imaging with single snapshot imaging of optical properties (SSOP)

**DOI:** 10.1007/s00464-024-11435-0

**Published:** 2024-12-04

**Authors:** Kohei Mishima, Marta Goglia, Luca Baratelli, Arturo Pardo, Giorgio Carlino, Riccardo Oliva, Simone Famularo, Ariosto Hernandez-Lara, Elisa Reitano, Pietro Riva, Alfonso Lapergola, Jacques Marescaux, Michel De Mathelin, Eric Felli, Sylvain Gioux, Michele Diana

**Affiliations:** 1 Research Institute Against Digestive Cancer (IRCAD), Strasbourg, France; 2 Department of Surgery, Keio University School of Medicine, Tokyo, Japan; 3 Department of Medical and Surgical Sciences and Translational Medicine, Faculty of Medicine and Psycology, Sapienza University of Rome, Rome, Italy; 4 Intuitive Surgical Sàrl, Aubonne, Switzerland; 5 IHU-Strasbourg, Institute of Image-Guided Surgery, Strasbourg, France; 6 Department of Digestive and Endocrine Surgery, Nouvel Hôpital Civil (NHC), Strasbourg University Hospital, Strasbourg, France; 7 University of Strasbourg, ICube Laboratory, Strasbourg, France; 8 Department of Visceral Surgery and Medicine, Inselspital, Bern University Hospital, University of Bern, Bern, Switzerland; 9 Department of Surgery, University Hospital of Geneva, Rue Gabrielle-Perret-Gentil 4, 1205 Geneva, Switzerland; 10 Medical Faculty, University of Geneva, Geneva, Switzerland

**Keywords:** Liver resection, Image-guided surgery, Single snapshot imaging of optical properties (SSOP), Liver ischemia

## Abstract

**Background:**

Identifying liver ischemia is crucial in liver surgery. This study aimed to develop a hemi-hepatic ischemia model for assessing liver ischemia using single snapshot imaging of optical properties (SSOP), a noninvasive optical imaging modality that provides real-time measurements of tissue oxygen saturation (StO2).

**Materials and methods:**

Twelve swine were randomly assigned to two groups: One undergoing total vascular inflow occlusion (TVIO) and the other undergoing hepatic artery occlusion (HAO). Preoperative 3D CT scans were used to locate the left-sided hepatic arteries and portal veins, which were clamped during surgery. Real-time SSOP imaging was conducted to measure StO_2_ in three lobes—the left lateral lobe (LL), left medial lobe (LM), and right medial lobe (RM)—as well as capillary lactate levels and Doppler blood flow. Measurements were recorded at baseline (T0), during ischemia (T1, 30 min after clamping), and during reperfusion (T2, 30 min after declamping).

**Results:**

In the TVIO group, SSOP imaging revealed a distinct demarcation line on the liver surface. StO_2_ levels measured by SSOP significantly decreased from T0 to T1, dropping by 29.8% in the LL (46.0 ± 5.1 vs. 16.2 ± 5.1%, p = 0.011) and 36.3% in the LM (42.7 ± 5.9 vs. 6.4 ± 4.0%, p = 0.001). Additionally, capillary lactate levels increased substantially in the LL (1.3 ± 0.4 vs. 8.5 ± 2.4 mmol/L, p = 0.041) and in the LM (1.3 ± 0.4 vs. 8.2 ± 2.1 mmol/L, p = 0.021). In contrast, the HAO group showed a less pronounced reduction in StO2: 13.6% in the LL (32.7 ± 6.4 vs. 19.1 ± 5.4%, p = 0.007) and 19.8% in the LM (35.3 ± 8.2 vs. 15.5 ± 5.8%, p = 0.011), with no significant increase in capillary lactate levels. An inverse correlation was found between StO_2_ and capillary lactate levels (r = − 0.76, p < 0.001).

**Conclusion:**

SSOP is a real-time, contrast-free imaging technique that effectively evaluates liver ischemia by accurately measuring tissue oxygenation, as validated by perfusion biomarkers.

**Supplementary Information:**

The online version contains supplementary material available at 10.1007/s00464-024-11435-0.

Identifying liver ischemia is crucial in liver resection (LR). In anatomical LR, the ischemic demarcation line serves as a key landmark for guiding parenchymal transection [1]. Additionally, the appropriate use of vascular occlusion techniques has been shown to significantly reduce intraoperative blood loss, particularly in cases involving large tumors or those located near major vessels [2]. However, assessing liver ischemia based solely on gross surface findings lacks objectivity, and Doppler ultrasound is limited by its inability to provide real-time intraoperative data during parenchymal transection. These limitations highlight the need for an objective, real-time diagnostic approach during LR.

To address these clinical demands, noninvasive imaging technologies have emerged at the forefront of medical research, with the potential to revolutionize liver surgery. One such modality is hyperspectral imaging (HSI), which has been utilized to assess hepatic oxygenation and differentiate between various models of liver ischemia [3], with successful clinical applications [4]. Our prior work integrated quantitative optical tissue characterization in HSI with artificial intelligence-based analysis, such as convolutional neural networks, to evaluate liver viability [5]. Although HSI has shown consistent and promising results, offering several applications compared to conventional methods [6, 7], it faces limitations such as the lack of video-rate capability, which restricts its use as a real-time surgical navigation tool.

In this study, we adopted single snapshot imaging of optical properties (SSOP) to evaluate liver ischemia. SSOP employs wide-field illumination to quantitatively determine tissue optical properties over a large field of view [8]. By leveraging deep learning networks, this technique enables real-time high-quality imaging of biological tissues [9]. We have previously documented the efficacy of SSOP-based tissue oxygenation assessments in evaluating perfusion during gastric tube formation in esophagectomy in pigs [10]. However, SSOP’s ability to evaluate liver ischemia has not been investigated. To explore this potential, we established a hemi-hepatic ischemia model to assess liver ischemia using SSOP-based oxygenation imaging. This approach mitigates systemic hemodynamic instability and reduces the impact of intestinal blood stasis associated with total hepatic inflow occlusion.

## Materials and methods

### Animals

This study received approval from the local ethical committee and was authorized by the French Ministry of Education, Research, and Innovation under Protocol notification (Approval number 28479–2,020,120,114,469,002). We used twelve female swine (Sus scrofa domesticus, 41–60 kg) as live models. These animals were randomly divided into two groups: One subjected to total vascular inflow occlusion (TVIO) and the other to hepatic artery occlusion (HAO).

In compliance with the European Directive 2010/63 and French animal protection laws, we ensured the ethical handling of animals in our laboratory. Prior to experimentation, the animals were group-housed and acclimatized in an enriched environment, which followed natural circadian light–dark cycles, with regulated humidity and temperature. The animals were pellet-fed twice a day (piglet diet Lorial/Costal 10–20 g/kg/day) and fasted 24 h before anesthesia with ad libitum access to water. All procedures took place under general anesthesia in a designated experimental operating room. Sedation for transfer to the operating room involved an intramuscular injection of azaperone (1–2 mg/kg, Stresnil® Elanco) and zolazepam + tiletamine (5–10 mg/kg, Zoletil® Virbac). Anesthesia induction was achieved with intravenous propofol (2–4 mg/kg, Propomitor®, Osalia) and rocuronium (1–2 mg/kg, Esmeron®, MSD), followed by orotracheal intubation. Maintenance of anesthesia used inhaled isoflurane at 2% (Isoflo®, Zoetis) in an Air/O_2_ mixture, with controlled ventilation provided by the Drager Primus® system. Monitoring included oximetry and heart rate throughout the procedure. Analgesia was administered via intravenous buprecare (0.01 mg/kg, Bupaq®, Virbac). At the end of the study, euthanasia was performed under anesthesia with an intravenous dose of pentobarbital (40 mg/kg IV, Euthoxin®, Osalia).

### 3-dimensional (3D) computed tomography (CT)

Prior to surgical intervention, we performed dynamic CT on all subjects. The arterial phase imaging started approximately 40 s after starting an auricular vein injection of a nonionic iodinated contrast medium (1 to 2 mL/kg Iomeprol, IOMERON®400, Bracco Imaging France) diluted in 20 mL of isotonic saline (NaCl 0.9%, Osalia), at an infusion rate of 1.5 to 2.5 mL/s. Subsequently, portal phase imaging was captured between 80 s and 2 min 35 s post-initial Iomeprol injection, followed by delayed phase imaging up to 7 min. The 3D Slicer software was used for 3D reconstruction.

### Surgical procedures

The pigs, positioned supine, underwent an upper midline laparotomy. The hepatoduodenal ligament was meticulously dissected to identify the hepatic arteries and portal veins, correlating with preoperative dynamic CT scans. The targeted vessels were clamped using Bulldog forceps (TVIO group: Left hepatic artery [LHA], middle hepatic artery [MHA], and left portal vein [LPV]; HAO group: LHA and MHA). Measurements of various parameters were taken at baseline (T0), during the ischemic phase (T1 [30 min after clamping]), and in the reperfusion phase (T2 [30 min after declamping]). Data were collected from three liver lobes: Left lateral (LL), left medial (LM), and right medial (RM). For further details, please refer to the supplementary file.

### Doppler ultrasonography

Doppler ultrasonography (ACUSON S3000 Ultrasound System, Healthineers, Germany) was used to measure blood flow velocities in the arteries and portal veins of each liver lobe, both before and after the clamping and declamping of vessels, ensuring adherence to the protocol.

### T-STAT tissue oximeter

Oxygen saturation levels within each liver lobe were measured for reference using a flap-type sensor on the t-STAT tissue oximeter (Spectros Medical Devices Inc., Texas, USA).

### Tissue lactate levels

Capillary lactate levels were determined using a strip-based portable lactate analyzer (Edge Lactate Test Strips, ApexBio Corp., Taiwan) in blood samples taken from the punctured Glisson’s capsule on the liver surface. We conducted a correlation analysis between SSOP-StO_2_ and capillary lactate levels to investigate the relationship between optical properties and hypoxic metabolism.

### 3D profile-corrected deep learning-based SSOP for measuring StO_2_

Spatial frequency domain imaging (SFDI) is an optical imaging technique based on the projection of spatially modulated light patterns onto the sample and based on the acquisition of the diffused backscattered light arising from it, using a camera. In its simplest configuration, SFDI requires at least six frames: two different spatial frequency patterns (e.g., fx = 0 mm^−1^, fx = 0.2 mm^−1^) and three-phase shifts for each of them. The phase-shifted sequence is then used to demodulate the signal, which allows to obtain the modulation amplitude maps of the given sample at each spatial frequency [11, 12]. A calibration step involving the measurement of a tissue mimicking phantom with known optical properties (absorption and reduced scattering coefficients), is used to compute the diffuse reflectance maps, i.e., RDC (fx = 0 mm^−1^) and RAC (fx = 0.2 mm^−1^). The pixel-wise optical properties of the sample are obtained through a Monte Carlo-based look-up-table (LUT) algorithm [11]. Due to the need for a minimum of six frames, SFDI cannot be used in real time. Conversely, single snapshot imaging of optical properties (SSOP) was recently developed to reduce the acquisition time and reach video-rate performances, thanks to a single high spatial frequency frame (i.e., fx = 0.2 mm^−1^) required to extract the optical properties of the sample [13]. In this study, we implemented the latest deep learning-based SSOP workflow for the demodulation of raw images, as described by Aguénounon et al*.* [9]. In short, the standard Fourier-domain filtering demodulation algorithm [13] was replaced by two dedicated U-Nets, the former being dedicated to the extraction of the modulation amplitude of the signal for each spatial frequency, and the latter being used for the profilometry analysis. Both networks were trained using high-quality images obtained with SFDI acquisition sequences (with 7-phase shifts instead of 3-phase ones to enhance quantification accuracy and image quality) and were optimized for real-time optical properties quantification for up to 1 MP images. The original network by Aguénounon et al*.* [9] was implemented in PyTorch as a monolithic PyTorch module, which was made up of two identical U-Nets (one for demodulation, and the other for phase extraction, each made up of one feature extraction branch and of two output branches). Following the exact specifications of Aguénounon et al*.* [9], the loss optimized by each network is the mean squared error (MSE) between the output of the model(s) and the ground truth provided by means of the SFDI. The optimizer used was Adam, with the same parameters as in the publication. The models were trained for 400000 iterations via stochastic gradient descent (batch size one) on full-resolution images. Lastly, a multiplicative learning rate scheduler was manually implemented to coincide with the one specified by the original article.

The training dataset consisted of a total of 407 high-quality images divided into various groups, namely tissue-mimicking silicone phantoms with different optical properties ranging from µa = 0.005 to 0.05 mm^−1^ for absorption and from µ’s = 0.5 to 3 mm^−1^ for reduced scattering; images of hands from different men and women in various poses, including wearing gloves; images of ex vivo porcine organs in several orientations (stomach, small bowel, colon, kidney, pancreas, liver, and spleen); images of ex vivo cow and pig muscle; images of fruits, vegetables, and plant leaves; and images of tools in various orientations. Brightness jitter was used to augment the training dataset as it is a well-known technique which ensures consistent inference under random exposure and illumination conditions [14]. Additionally, 3D profilometry corrections of the sample were used to reduce the quantification error of SSOP associated with a variation of light intensity across non-flat sample surfaces. Oxygenation computation was achieved by applying Lambert–Beer’s law for chromophore absorption inside the biological tissue [15]. In addition to static images captured at three timepoints (i.e., baseline (T0), during the ischemic phase (T1, [30 min after clamping]), and in the reperfusion phase (T2, [30 min after declamping]), blood flow dynamics in the liver was evaluated intermittently by recording videos before and after vessel clamping and declamping.

### Instrumentation

The imaging system used for the study has been described [16]. The device included a white light plasma lamp (HPLS301, Thorlabs, Inc., Newton, NJ, USA) for the illumination of the surgical field, together with a fiber-coupled custom-made high-power two-wavelength laser source working at 665 and 860 nm. The projection of sinusoidal patterns on the sample was performed using a Digital Micromirror Device-based projector (V-7001, Vialux GmbH, Chemnitz, Sachsen, Germany). The working distance of the imaging head was kept at 45 cm from the surgical field, subsequently determining a field-of-view (FOV) of 15 × 15 cm while still providing a comfortable operating condition for the surgeon. The imaging head was based on a 3-channel architecture. Two near-infrared monochrome cameras (PCO.edge 4.2 and GO-5000 M-USB, JAI Ltd., Kanagawa, Japan) were used for the acquisition of the SSOP frames with 1,024 × 1,280 pixel resolution at 665 and 860 nm, and an RGB camera (GO-5000C-USB, JAI Ltd., Kanagawa, Japan) was also added to record the surgical field. The co-registration of the scene via the cameras was obtained thanks to a customized optomechanical system in which optical filters were included to separate the 3 channels at the collection side. In addition, a pair of linear polarizers (PPL05C, Moxtek, Orem, UT, USA) were used in a crossed configuration at the projection and camera sides, to reject the specular reflections originating from the sample surface.

### Statistical analysis

Two-way ANOVA was used to analyze differences between the LL, LM and RM groups (GraphPad Prism 10.2.2). Pearson’s correlation matrix was used to understand the correlation between optical and biological parameters. P value < 0.05 was considered significant.

## Results

**Fig. 1:**
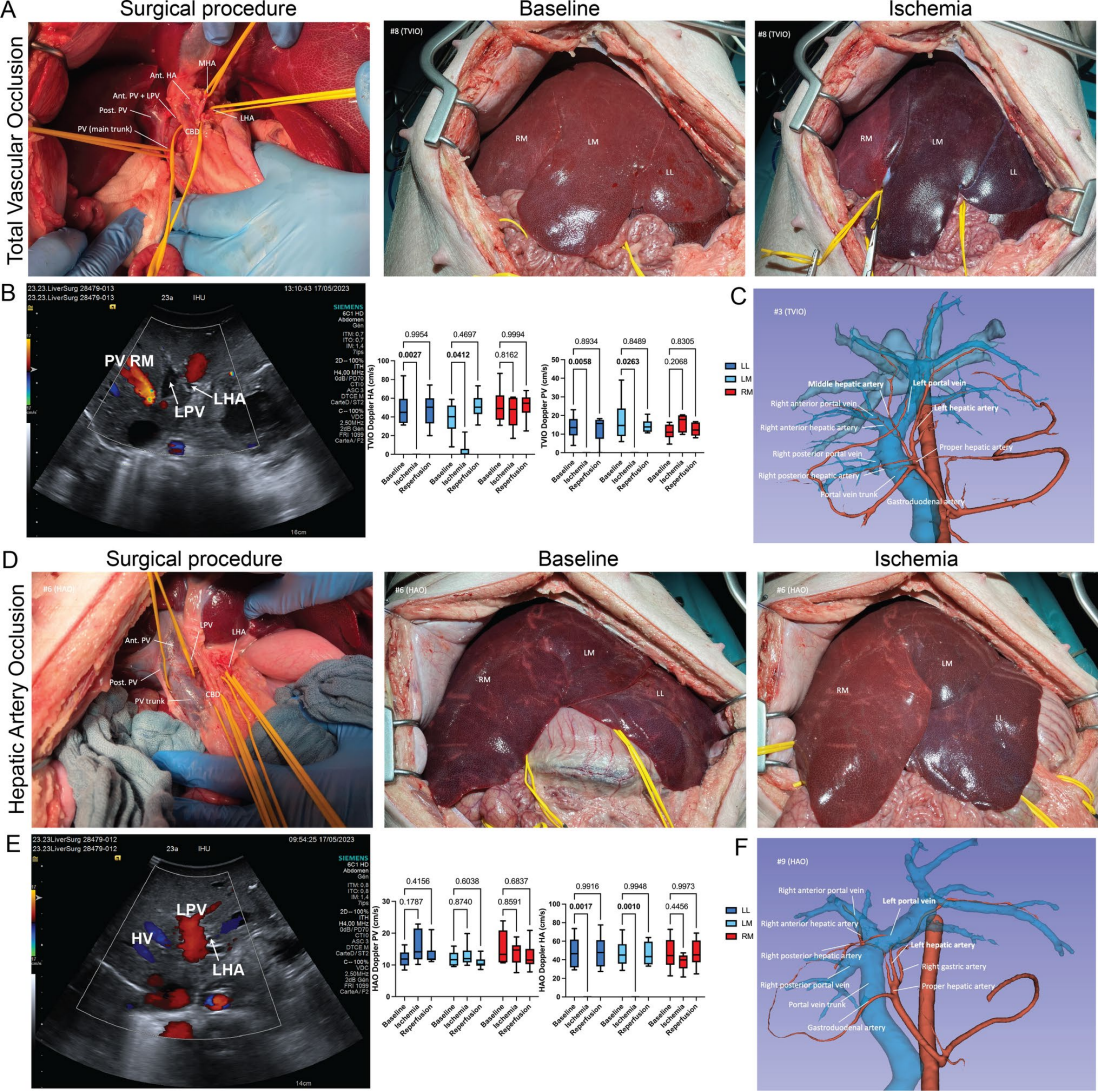
Visual results of surgical procedure. **A** Vascular structure in the hepatoduodenal ligament and liver surface at baseline and under ischemia in the TVIO group. **B** Doppler ultrasonography of the liver blood flow. **C** Preoperative 3D CT images of the vascular anatomy. **D** Vascular structure in the hepatoduodenal ligament and liver surface at baseline and under ischemia in the HAO group. **E** Doppler ultrasonography of the liver blood flow. **F** Preoperative 3D CT images of the vascular anatomy. *LHA* left hepatic artery, *MHA* middle hepatic artery, *LPV* left portal vein, Ant. *HA* right anterior hepatic artery, Ant. *PV* right anterior portal vein, Post. Portal vein = right posterior portal vein, *PV* portal vein, *CBD* common bile duct, *LL* left lateral lobe, *LM* left medial lobe, *RM* right medial lobe. ACUSON S3000 Ultrasound System (Healthineers, Germany) was used for Doppler ultrasonography. Two-way ANOVA multiple comparison was used to analyze differences between the groups. P value < 0.05 was considered statistically significant

TVIO and HAO were performed in 12 pigs, divided equally into two groups. In the assessment of left-sided hepatic arteries (HAs), the MHA was identified in 9 pigs, originating from the right anterior hepatic artery (Aant) in 5 pigs, the LHA in 3 pigs, and the proper hepatic artery (PHA) in 1 pig. A solitary LHA pattern was observed in 1 pig, while an early branching pattern involving laterosuperior (A2), lateroinferior (A3), and medial segment (A4) arteries was noted in 2 pigs. Concerning the portal vein (PV), a common trunk combining the left and right anterior (LPV/Pant) PV pattern was found in 11 pigs (Supplementary Materials in Table 1). For the TVIO group, clamping was applied to the LHA, MHA (or A2, A3, and A4), and LPV. In the HAO group, only the LHA and MHA were clamped (Supplementary Materials in Table 1). In the TVIO group (Fig. 1A), vascular clamping resulted in a distinct demarcation line on the liver surface. Doppler ultrasound confirmed the cessation of both arterial and portal blood flow in the LL (HA: 48.4 ± 7.7 vs. 0.0 ± 0.0, p = 0.003, PV: 13.6 ± 2.6 vs. 0.0 ± 0.0, p = 0.006) and LM lobes (HA: 38.5 ± 7.2 vs. 4.0 ± 4.0, p = 0.041, PV: 7.2 ± 4.7 vs. 0.0 ± 0.0, p = 0.026) at T1 (Fig. 1B). The anatomical details derived from 3D-CT imaging were referenced throughout the procedure (Fig. 1C). Conversely, in the HAO group (Fig. 1D), the demarcation line was less pronounced and varied in intensity. At T1, arterial flow cessation was observed in the LL (HA: 47.7 ± 6.9 vs. 0.0 ± 0.0, p = 0.002, PV:12.0 ± 1.1 vs. 15.7 ± 2.1, p = 0.179) and LM (HA: 46.8 ± 6.0 vs. 0.0 ± 0.0, p = 0.001, PV: 12.0 ± 1.0 vs. 12.9 ± 1.5, p = 0.874) at T1 (Fig. 1E). The 3D-CT images provided continuous anatomical guidance during the procedure (Fig. 1F).

**Fig. 2:**
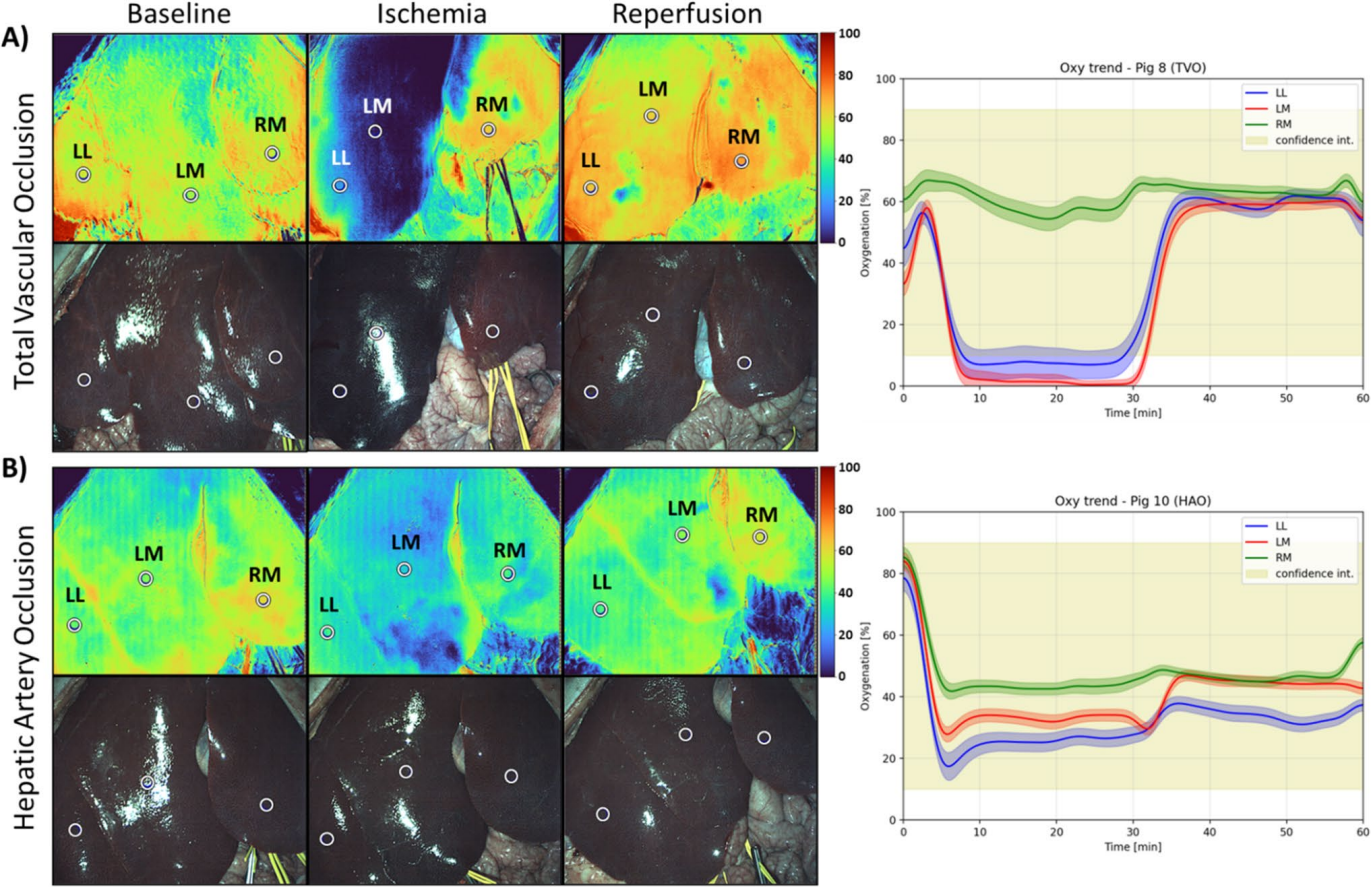
SSOP imaging and SSOP-StO2. **A** Total vascular inflow occlusion. **B** Hepatic artery occlusion. Left: SSOP images during baseline (T1 = 0 min), ischemic (T2 = 30 min post-clamping), and reperfusion phases (T2 = 30 min post-declamping). Right: SSOP-StO2 changes over time (0–60 min). Clamping of the left hepatic artery and portal vein resulted in a clear color change on SSOP imaging in the left liver and a consequent reduction in StO_2_. Clamping only the left hepatic artery showed only mild SSOP imaging changes and a mild decrease in StO_2_ in the left liver. *LL* Left lateral lobe, *LM* Left medial lobe, *RM* Right medial lobe, *Conf Int* = Confidence interval

SSOP imaging was performed throughout the experiment to measure SSOP-based StO_2_ in each liver lobe (LL, LM, RM). In the TVIO group, SSOP imaging revealed a pronounced ischemic demarcation line, with the SSOP-StO_2_ curve showing a steep reduction in StO_2_ in both LL and LM after vascular clamping. StO_2_ levels remained consistently low during the ischemic phase and promptly returned to baseline following declamping (Fig. 2A). In contrast, the HAO group exhibited subtler changes in StO_2_, as indicated by SSOP imaging andStO_2_ measurements in the LL and LM (Fig. 2B).

**Fig. 3:**
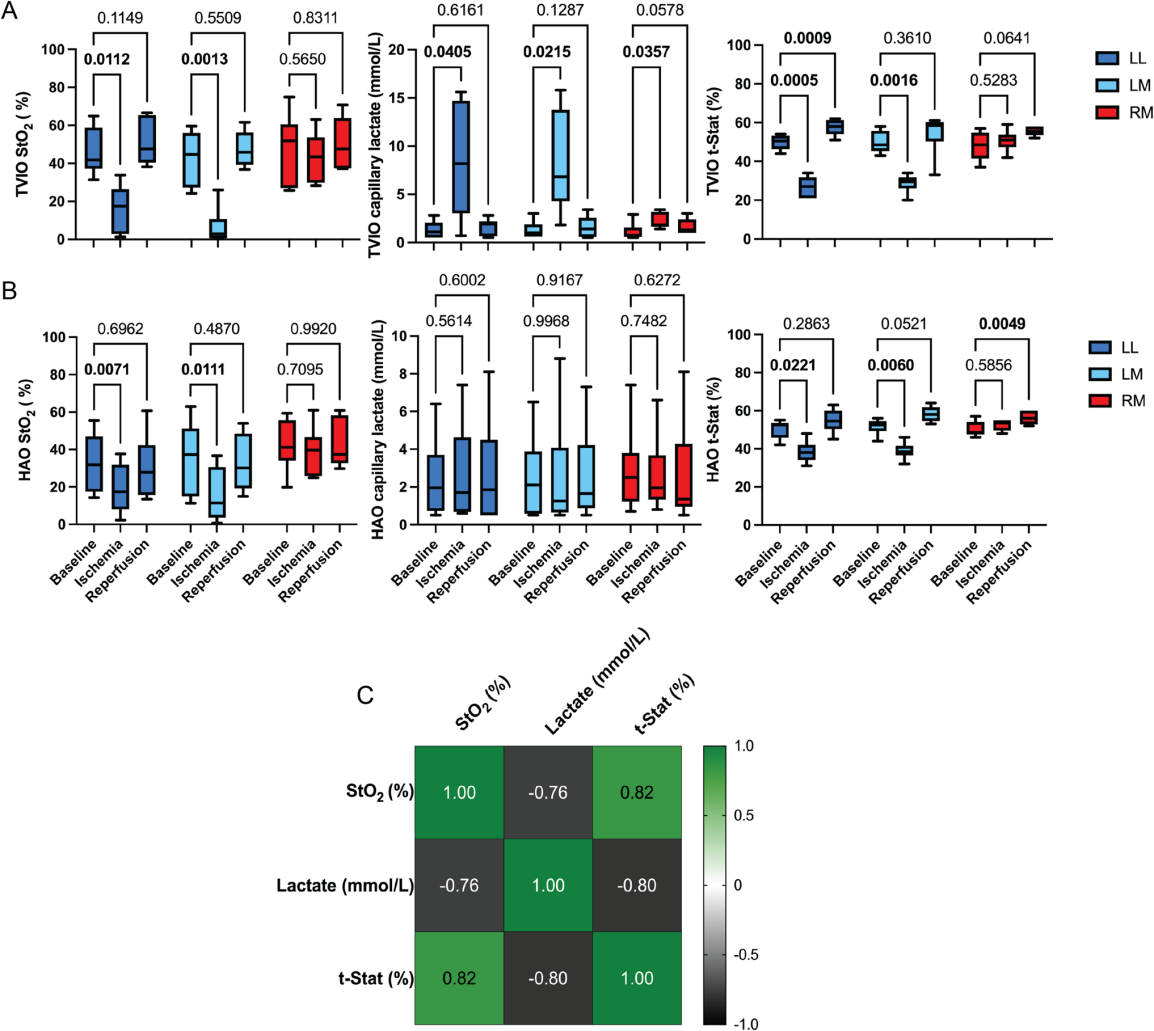
Statistical analysis of 12 cases. **A** StO_2_, capillary lactate levels, and t-Stat values in the TVIO group, **B** StO_2_, capillary lactate levels, and t-Stat in the HAO group, **C** Correlation analysis. *LL* Left lateral lobe, *LM* Left medial lobe, *RM* Right medial lobe. Two-way ANOVA multiple comparison was used to analyze differences between groups. Pearson’s correlation coefficient was used to analyze correlation between parameters. P value < 0.05 was considered statistically significant

In the TVIO group (Fig. 3A), StO_2_ decreased significantly by 29.8% in the LL (46.0 ± 5.1 vs. 16.2 ± 5.1%, p = 0.011) and by 36.3% in the LM (42.7 ± 5.9 vs. 6.4 ± 4.0%, p = 0.001), while the RM did not show a significant change (48.0 ± 7.7 vs. 43.2 ± 5.3%, p = 0.565). Capillary lactate levels increased markedly in the LL (1.3 ± 0.4 vs. 8.5 ± 2.4 mmol/L, p = 0.041) and LM (1.3 ± 0.4 vs. 8.2 ± 2.1 mmol/L, p = 0.021), with a more modest rise in the RM (1.1 ± 0.4 vs. 2.2 ± 2.4 mmol/L, p = 0.036). T-Stat-StO_2_ values decreased by 23.0% in the LL (49.8 ± 1.6 vs. 26.8 ± 2.2%, p < 0.001) and by 21.1% in the LM (49.8 ± 2.4 vs. 28.7 ± 1.9%, p = 0.002), while remaining unchanged in the RM (48.0 ± 3.0 vs. 50.7 ± 2.2%, p = 0.528).

In the HAO group (Fig. 3B), StO_2_ decreased by 13.6% in the LL (32.7 ± 6.4 vs. 19.1 ± 5.4%, p = 0.007) and by 19.8% in the LM (35.3 ± 8.2 vs. 15.5 ± 5.8%, p = 0.011), with no significant change in the RM (42.5 ± 5.7 vs. 38.9 ± 5.4%, p = 0.701). Capillary lactate levels remained stable in the LL (2.4 ± 0.9 vs. 2.6 ± 1.1 mmol/L, p = 0.561), LM (2.5 ± 1.3 vs. 2.5 ± 1.3 mmol/L, p = 0.997), and RM (2.9 ± 1.0 vs. 2.6 ± 0.8 mmol/L, p = 0.748). T-Stat-StO_2_ values decreased by 12.0% in LL (50.3 ± 1.9 vs. 38.3 ± 2.3%, p = 0.022) and by 12.9% in the LM (51.7 ± 1.7 vs. 38.8 ± 1.8%, p = 0.002), while remaining unchanged in the RM (50.2 ± 1.7 vs. 52.5 ± 1.2%, p = 0.586). Correlation analysis revealed that SSOP-based StO_2_ levels were positively correlated with T-Stat-StO_2_ values (r = 0.82, p < 0.001) and inversely correlated with tissue lactate levels (r = -0.76, p < 0.001) (Fig. 3C).

## Discussion

This study evaluated the utility of SSOP in a hemi-hepatic ischemia model using twelve pigs, which were divided into two groups: TVIO and HAO. Preoperative 3D-CT scans confirmed the extrahepatic vascular anatomy, and intraoperative Doppler ultrasonography verified the occlusion of targeted vessels, ensuring procedural accuracy. The dynamics of SSOP imaging were monitored through video recordings and still images at baseline, ischemia, and reperfusion phases.

In the TVIO group, StO_2_ in the left liver decreased sharply after vessel clamping and remained consistently low throughout the ischemic phase. In contrast, the HAO group showed a more moderate decline in StO_2_, indicating partial preservation of oxygen saturation in the liver parenchyma due to residual portal venous flow. Notably, a significant elevation in capillary lactate levels was observed only in the TVIO group, suggesting that in the HAO group, hepatocyte oxygenation was maintained by both portal and collateral arterial blood flow. Additionally, in the TVIO group, elevated capillary lactate levels in the RM during the ischemic phase likely indicated that lactate generated by prolonged ischemia in the left liver was entering the systemic circulation via the hepatic vein, leading to increased systemic lactate levels.

The findings of this study indicate that SSOP-based StO2 is closely correlated with capillary lactate levels and t-Stat-StO2, capturing a unified physiological response to liver ischemia. StO2, which provides real-time monitoring without the need for dedicated imaging intervals, could potentially replace spot measurements from other modalities. Although the lack of pathological histology precludes definitive conclusions about changes in hepatic lobule ultrastructure, previous studies using HSI and SSOP have extensively addressed this aspect [3, 5, 10].

Minimally invasive liver surgeries, such as laparoscopic and robotic LRs, are particularly well-suited to benefit from SSOP technology. In laparoscopic anatomical LR, the demarcation line on the liver surface serves as a crucial landmark [17]. However, in cases where the liver parenchyma is rough, such as with cirrhosis, identifying this line can be challenging. Although fluorescence-guided surgery using indocyanine green (ICG) is gaining popularity [18], backflow in the portal or hepatic veins can obscure the boundary. SSOP imaging offers a complementary solution by providing clear visualization of ischemic changes during anatomical LR.

Another promising application of SSOP lies in its use of quantitative StO2 data, which can optimize LR procedures by correlating intraoperative liver ischemia with clinical outcomes. Traditionally, the impact of surgical maneuvers on blood loss and patient prognosis has been studied using Pringle’s maneuver duration as a key parameter. However, real-time monitoring of SSOP-StO2 during parenchymal transection could enable the identification of new surrogate markers for perioperative complications and long-term outcomes.

Future studies could expand on this work by incorporating intermittent, whole-liver inflow occlusion (Pringle’s maneuver), as well as partial hepatic vein occlusion and venous congestion, as described in HSI studies [19]. Such an approach would enable a more comprehensive assessment of the remnant liver’s response during major hepatectomies or liver transplantations, utilizing SSOP-StO_2_ measurements alongside other parameters, as previously investigated with HSI [20, 21].

## Conclusions

SSOP-StO_2_ is a reliable, real-time, and contrast-free method for assessing liver ischemia, effectively identifying tissue oxygenation as validated by perfusion biomarkers.

## Supplementary Information

Below is the link to the electronic supplementary material.

Supplementary file1 (DOCX 16 KB)
