# Supplementary material for: Quantification of hemi-hepatic ischemia using real-time multispectral oxygenation imaging with single snapshot imaging of optical properties (SSOP)
Source: Surg Endosc. 2024 Dec 4;39(2):898–906. doi: 10.1007/s00464-024-11435-0 (PMC11794345; doi:10.1007/s00464-024-11435-0)
Supplement: Supplementary file 1 — Supplementary file1 (DOCX 16 KB) [file 464_2024_11435_MOESM1_ESM.docx]

# Supplementary materials

**Surgical Procedure**

After laparotomy, the lesser omentum was open to confirm the caudate lobe. Then, the portal vein (PV) and the common bile duct (CBD) were dissected from the right side and taped. Next, the left hepatic artery (LHA) and middle hepatic artery (MHA) were dissected from the ventral side and taped. The CBD and hepatic arteries were ventrally retracted, the ventral side of the common trunk of the left portal vein (LPV) and right anterior PV (Pant) was dissected and its bifurcation was confirmed. A gauze was placed into both the right and left subphrenic spaces, and a stitch was added on the right upper quadrant and retracted to the right for better exposure of the three targeted liver lobes. Finally baseline parameters were sampled (T0), including SSOP, T-Stat, capillary lactate, and Doppler ultrasonography. LPV and LHA/MHA were clumped with Bulldog forceps to confirm blood flow with Doppler ultrasonography. Measurements were made 30 minutes after clamping (T1) and then 30 minutes after declamping (T2).

**Table 1** Extrahepatic vascular anatomies in 12 pigs

| No. | Group | Left-sided HAs | Left-sided PV | Clamped vessels |
| --- | --- | --- | --- | --- |
| 1 | TVIO | LHA, MHA from Aant | LPV/Pant | LHA, MHA, LPV |
| 3 | TVIO | LHA, MHA from Aant | LPV/Pant | LHA, MHA, LPV |
| 4 | HAO | LHA (→MHA) | LPV/Pant | LHA, MHA |
| 6 | HAO | LHA, MHA from Aant | LPV | LHA, MHA |
| 7 | TVIO | LHA, MHA from PHA | LPV/Pant | LHA, MHA, LPV |
| 8 | TVIO | LHA, MHA from Aant | LPV/Pant | LHA, MHA, LPV |
| 9 | HAO | LHA (→A2, A3, A4) | LPV/Pant | A2, A3, A4 |
| 10 | HAO | LHA (→A2, A3, A4) | LPV/Pant | A2, A3, A4 |
| 11 | TVIO | LHA (→MHA) | LPV/Pant | LHA, MHA, LPV |
| 12 | HAO | LHA (→MHA) | LPV/Pant | LHA, MHA |
| 13 | TVIO | LHA | LPV/Pant | LHA, LPV |
| 14 | HAO | LHA, MHA from Aant | LPV/Pant | LHA, MHA |

Table1. Extrahepatic vascular anatomies in 12 pigs. TVIO=total vascular inflow occlusion, HAO=hepatic artery occlusion, LHA=left hepatic artery, MHA=middle hepatic artery, Aant=right anterior hepatic artery, A2=laterosuperior segment artery, A3=lateroinferior segment artery, A4=left medial lobe artery, LPV= left portal vein, Pant=right anterior portal vein.
